# Comparing malaria early detection methods in a declining transmission setting in northwestern Ethiopia

**DOI:** 10.1186/s12889-021-10850-5

**Published:** 2021-04-24

**Authors:** Dawn M. Nekorchuk, Teklehaimanot Gebrehiwot, Mastewal Lake, Worku Awoke, Abere Mihretie, Michael C. Wimberly

**Affiliations:** 1 Department of Geography and Environmental Sustainability, University of Oklahoma, Norman, OK USA; 2 Amhara Public Health Institute, Bahir Dar, Ethiopia; 3 School of Public Health, Bahir Dar University, Bahir Dar, Ethiopia; 4 Health, Development, and Anti-Malaria Association, Addis Ababa, Ethiopia

**Keywords:** Malaria, Early detection, Event detection, Farrington algorithm, Ethiopia, *Plasmodium falciparum*

## Abstract

**Background:**

Despite remarkable progress in the reduction of malaria incidence, this disease remains a public health threat to a significant portion of the world’s population. Surveillance, combined with early detection algorithms, can be an effective intervention strategy to inform timely public health responses to potential outbreaks. Our main objective was to compare the potential for detecting malaria outbreaks by selected event detection methods.

**Methods:**

We used historical surveillance data with weekly counts of confirmed *Plasmodium falciparum* (including mixed) cases from the Amhara region of Ethiopia, where there was a resurgence of malaria in 2019 following several years of declining cases. We evaluated three methods for early detection of the 2019 malaria events: 1) the Centers for Disease Prevention and Control (CDC) Early Aberration Reporting System (EARS), 2) methods based on weekly statistical thresholds, including the WHO and Cullen methods, and 3) the Farrington methods.

**Results:**

All of the methods evaluated performed better than a naïve random alarm generator. We also found distinct trade-offs between the percent of events detected and the percent of true positive alarms. CDC EARS and weekly statistical threshold methods had high event sensitivities (80–100% CDC; 57–100% weekly statistical) and low to moderate alarm specificities (25–40% CDC; 16–61% weekly statistical). Farrington variants had a wide range of scores (20–100% sensitivities; 16–100% specificities) and could achieve various balances between sensitivity and specificity.

**Conclusions:**

Of the methods tested, we found that the Farrington improved method was most effective at maximizing both the percent of events detected and true positive alarms for our dataset (> 70% sensitivity and > 70% specificity). This method uses statistical models to establish thresholds while controlling for seasonality and multi-year trends, and we suggest that it and other model-based approaches should be considered more broadly for malaria early detection.

**Supplementary Information:**

The online version contains supplementary material available at 10.1186/s12889-021-10850-5.

## Background

Over the last few decades, incredible progress has been made worldwide in reducing malaria cases and deaths. From 2010 to 2018, the incidence of malaria cases declined globally from 71 to 57 cases per 1000 population at risk and malaria deaths fell by 31% during the same period [1]. However, between 2014 and 2018, the decreasing trend in incidence flattened with some regions seeing an increase in cases, indicating stalled progress. In 2018, there were an estimated 228 million cases worldwide, which was 3 million less than 2017, but 1 million more than in 2016 [1]. Of the total cases in 2018, 93% (213 million) occurred in the World Health Organization (WHO) African region [1]. In addition, due to population growth, the absolute number of people at risk for malaria globally is increasing, with the sharpest increase seen in the WHO African region [2]. As a result, there is a continuing need for improved strategies and tools to support malaria prevention, control, and elimination.

Malaria surveillance as a core intervention strategy is one of the pillars of the Global Technical Strategy for malaria [3, 4]. Information from surveillance systems can be used to optimize interventions to interrupt disease transmission and ultimately accelerate elimination. Timely detection allows officials to intensify control measures as needed to manage epidemics [4–10]. Many early-detection algorithms exist, and there is a need to quantitatively evaluate the performance of these algorithms for different diseases and locations [11–17]. The central idea behind outbreak detection is to identify when the case volume exceeds a baseline threshold, and to use this information in a prospective (not retrospective) manner to identify epidemics in their early stages [4, 15]. Various algorithms are used to calculate these thresholds, with different assumptions about the pattern of disease transmission, including the speed of outbreak development, seasonality, and trends [10, 12–16, 18–30].

Early detection algorithms that have been proposed for malaria include Cullen, WHO quartile, and cumulative sum (CUSUM) methods [4, 5, 10, 12, 17, 19]. These techniques define thresholds based on statistical summaries of historical data. The Cullen and quartile methods are recommended by WHO to have at least five years of past data to generate reliable estimates of the thresholds [5, 12]. The Cullen method calculates the mean value over the past five years of the current time period (e.g., week or month of year), excluding values from any past outbreak periods. Case volumes over the mean plus two standard deviations are considered outbreaks [5, 12, 20]. The WHO quartile method defines an outbreak by calculating quartile values for the current seasonal time period (usually the week or month of the year) over the past five years. An outbreak is identified when cases exceed the third (75%) quartile. This approach may be sensitive to slight increases in case volume during time periods when there have never been spikes or outbreaks of cases, but is less affected by abnormal years compared to the Cullen method [5, 12]. Several variations of the statistical methods have been evaluated from selected health center data in Ethiopia, and weekly percentile measures performed as well as ones with more complex threshold calculations [17]. There are many variations of the cumulative sum (CUSUM) approach, a type of control chart that tracks cumulative differences between observed values and expected values and indicates an outbreak when these cumulative differences exceed a threshold [5, 12, 18, 21–23].

In many situations, sufficient historical data may not be available to implement these approaches. Even when historical data are available, older data may be more representative of past malaria transmission cycles than the current malaria situation [4, 10]. In places undergoing intensive malaria intervention efforts, incidence in recent years may be significantly reduced compared to only a few years in the past or may exhibit different seasonal patterns [25]. Thresholds based on previous years may then fail to capture the new patterns and intensities of current outbreaks. However, surveillance and outbreak detection are still crucial in areas of low or unstable transmission. Immunity to malaria decreases with the intensity of malaria transmission, and the population could be highly vulnerable to malaria outbreaks [5].

Other early detection algorithms use different approaches for the calculation of the thresholds and may be more applicable in regions undergoing rapid change in malaria transmission patterns. The CDC Early Aberration Reporting System (EARS) has been used as a drop-in technique for syndromic surveillance after major incidents that could precipitate disease outbreaks [16, 22, 26, 27, 31]. This suite of methods is similar to quality control charts and relies on only very recent data to create a baseline and is therefore useful when long-term data is not available or not relevant to the current situation. The EARS system is actively used by U.S. state and local public health offices [26]. Syndromic surveillance using school-based absenteeism has been investigated for potentially identifying localized malaria outbreaks in Ethiopia [32]. A family of methods developed by Farrington and later, Noufaily, have been implemented at several European infectious disease control centers [24, 28, 33–35]. Farrington methods are based on quasi-Poisson regression and can take advantage of historical information while accounting for seasonality, long-term trends, and previous outbreaks [24, 28, 29].

While previous research has compared various detection algorithms, many of these studies have used simulated datasets (e.g. [16, 22, 30]), and it is unclear the extent that these would be representative of real world outbreaks, especially in the context of public health interventions. Therefore, in this article, we used a 7.5 year weekly surveillance dataset of malaria cases to test the suitability of 1) CDC EARS methods, 2) methods based on weekly statistical thresholds (including WHO and Cullen methods), and 3) Farrington methods to detect malaria outbreaks. To develop a baseline dataset of malaria outbreaks, we applied a novel method to identify malaria events of interest to use as retrospective test cases. This research was conducted in the Amhara region of Ethiopia, which has been the subject of intense malaria interventions and experienced a general decline in malaria cases [36]. In 2019 there was a resurgence of malaria cases in the region, and we used this year as the basis for testing the outbreak detection algorithms. Our main objectives were to compare sensitivity and true positive rates of the event detection methods applied to malaria outbreak detection and to assess their potential for detecting outbreaks.

## Methods

### Study area and data

**Fig. 1:**
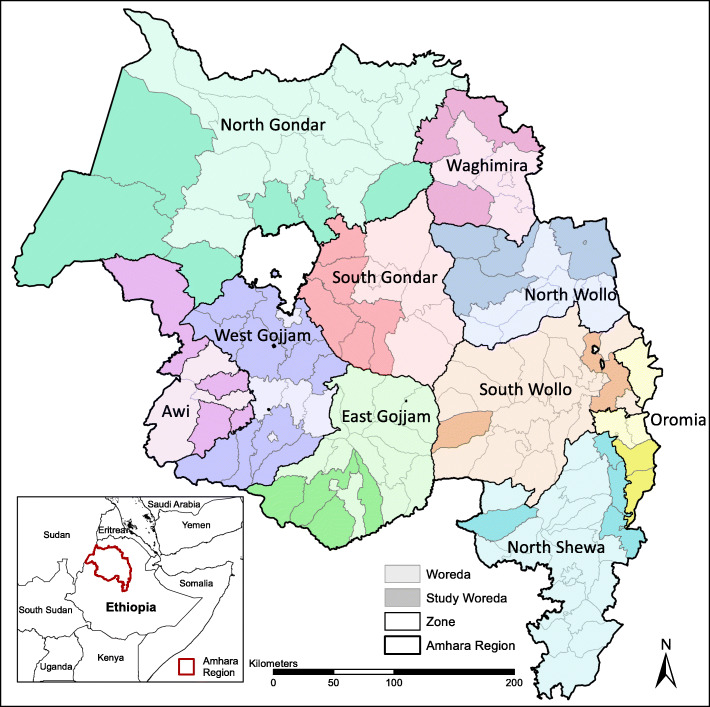
Amhara region of Ethiopia. Zones are labeled and the 47 woredas included in this study are marked in darker shades

The Amhara region is located in northwest Ethiopia (Fig. 1). Most of the terrain is mountainous, with lowlands along the northwestern edge of the region. Rainfall is highly seasonal, with the heaviest rains from June through September. There are two major seasons for malaria transmission: the main transmission season after the end of the rainy season between September and December, and a secondary peak at the beginning of the rainy season in May through August [37, 38]. Population in the Amhara region is over 21 million, and the people primarily live in rural areas and practice subsistence farming [39]. There is widespread transmission of *Plasmodium falciparum* and *P. vivax* malaria, with a ratio of 1.2 of *P. falciparum* to *P. vivax* as seen in blood film tests from a cross-sectional survey [40]. A national malaria control program targets the Ethiopian population at risk, including the Amhara region. The program includes four main interventions: distribution of free long-lasting insecticidal nets (LLINs), indoor residual spraying (RDS), rapid diagnostic tests (RDTs) available at all health facilities, and treatment with artemisinin-based combination therapy (ACT) [39, 40]. Areas with low transmission rates due to declining malaria incidence and unstable transmission patterns are being targeted for elimination [39, 41, 42].

Administratively, the region is divided into twelve zones and three administered towns, which are further divided into between four and 24 woredas, or districts (Fig. 1). Woredas are subdivided into kebeles (villages). In the Amhara region, there are 162 woredas (containing 3543 kebeles), and 47 of the most malaria-prone woredas were included in the Epidemic Prognosis Incorporating Disease and Environmental Monitoring for Integrated Assessment (EPIDEMIA) pilot project [39]. The health care system is organized into three tiers: primary, secondary, and tertiary levels [43]. The primary level in rural areas includes health posts, health centers, and a primary hospital. The primary health care units (PHCUs) contain five health posts (satellite facilities located in kebeles) and one referral health center. Secondary and tertiary levels are referral general and specialized hospitals, respectively.

Public health surveillance data on patients seeking care at health posts or health centers are collected and aggregated by the Amhara Regional State Health Bureau (ARHB). Among the data collected are the numbers of malaria cases confirmed by rapid-diagnostic tests (RDT) or blood film screening, and these counts are grouped as *Plasmodium falciparum* (including mixed infections) and *P. vivax* (only) malaria. These data are summarized by the week of the year (based on the ISO 8601 standard used by WHO) and reported to the woreda health office. This office aggregates a complete woreda report before sending the summarized data to the zonal health office, which compiles all the woreda reports within the zone and sends the reports to the regional ARHB office, where they were uploaded into the EPIDEMIA system [39].

**Fig. 2:**
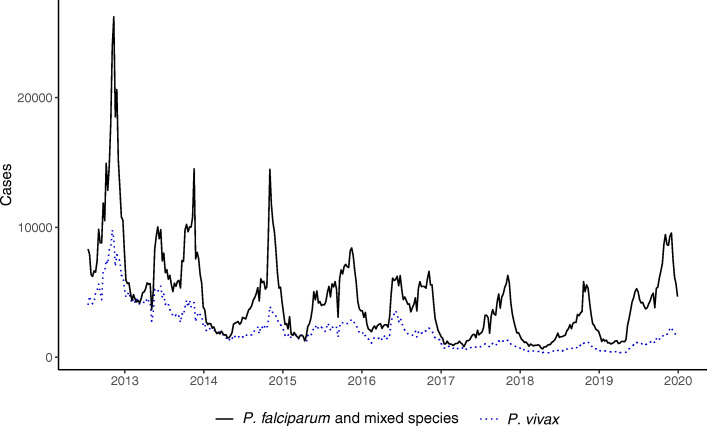
Time series graph of malaria case counts by species. The graph of *Plasmodium falciparum* (and mixed species) and *P. vivax* case counts shows the patterns of seasonality, long-term declining trends, and resurgence in 2019

This study analyzed data from the 47 EPIDEMIA pilot woredas, which included weekly case counts of *P. falciparum* (or mixed) and *P. vivax* malaria starting from ISO week 28 of 2012 through week 52 of 2019. These woredas have seen great public health successes in reducing the malaria burden from 2012 through 2018, but experienced a resurgence in 2019 (Fig. 2).

Between 2013 and 2018, there was a steady decrease from 349,523 *P. falciparum* or mixed malaria cases to 104,947 cases, a 70% reduction. However, in 2019 there were 210,194 cases, a volume that had not been seen since 2015 (Table 1). We focused our analysis on *P. falciparum* (including mixed infections with *P. vivax*) which is the predominant parasite species, is of greatest concern from a public health standpoint, and had the strongest resurgence in 2019.

**Table 1:** Confirmed malaria case counts from 47 pilot woredas in the Amhara region by species

Year	*P. falciparum* and mixed	*P. vivax*	Total
*2012 (W28-W52)*	*306,832*	*157,983*	*464,815*
2013	349,523	208,861	558,384
2014	216,553	108,989	325,542
2015	222,103	110,746	332,849
2016	203,610	98,146	301,756
2017	125,415	45,176	170,591
2018	104,947	31,695	136,642
2019	210,194	51,906	262,100

### Identification of baseline events via trend weighted seasonal thresholds (TWST)

Prior to evaluating event detection algorithms, specific events of interest must be defined for each woreda to be the baseline testing dataset. Here, for research purposes, we developed an objective approach named trend weighted seasonal thresholds (TWST) for identifying events as anomalous increases in the number of reported malaria cases. The approach was designed to identify events retrospectively in the context of seasonal patterns and decreasing long-term trends in disease transmission, while allowing for variation in patterns across woredas as well as slight time-shifts in seasonal peaks between years.

The TWST approach identified two thresholds, weekly and yearly, for each woreda. This combination of weekly and yearly thresholds has been used in other work for defining malaria epidemics [13]. In preparation, the raw weekly time-series were smoothed using a centered 5-week triangular moving average, which used a sliding window of five weeks with the week being calculated in the center. The moving average was weighted with the center week the most important, and the weeks on either side having decreasing weight. The yearly threshold was calculated as the harmonic mean of the entire year plus a multiplier based on the standard deviation (1.5 for *P. falciparum* and mixed species).

The weekly threshold was calculated in a three-step process. In the first step, the raw threshold value for a given week was the harmonic mean of that week in the year, over the five years of data, plus a multiplier based on the standard deviation (1 for *P. falciparum* and mixed). In the second step, the raw thresholds were optionally trend weighted based on the year harmonic mean. If there was a declining trend (from the year previous), then the weekly threshold values were weighted proportional to the difference between the current year harmonic mean and the highest (max) harmonic mean using a weighting factor, 0.5 for *P. falciparum* and mixed: (max – weighting factor * (max – current)) / max. If there was no declining trend, the weekly thresholds were weighted based on the previous year mean instead of the current year. In the third and final step, allowances were made to prevent minor time shifts in increasing and decreasing case counts between years from triggering alerts [32, 44], by inflating weekly thresholds that were not near in time to peaks. Peak areas were identified using a percentile cut-off per year (85% for *P. falciparum* and mixed), plus short stretches (up to 8 weeks) between these high rates. The inflation was based on the average of the year and week harmonic means multiplied by an expansion factor (1.2 for *P. falciparum* and mixed), which was then added to the trend weighted threshold of the previous step to arrive at the final TWST week threshold. Anomalies were identified if cases exceeded both the yearly and weekly thresholds, and events were identified if anomalies lasted for four or more weeks consecutively. Events that were separated by only one or two weeks were merged into one event.

### Event detection

#### Detection algorithms

The previous step, event identification, was based on a retrospective analysis with full knowledge of the entire 7.5-year time span, yielding specific spikes or abnormal increases in malaria case counts to be used as a baseline testing set for the detection algorithms. In contrast, event detection algorithms were forward-looking, running in-step with the data and only using values up to a given week, which mimics real time surveillance efforts to detect outbreaks as early as possible to mount timely public health responses. For this study, three types of event detection algorithms were used: 1) CDC EARS methods, 2) weekly statistical summaries that included the commonly-used WHO and Cullen methods, and 3) Farrington methods [4, 27–29].

For EARS, the three variations C1-Mild, C2-Medium, and C3-High were tested using the default alpha values (0.001 for C1 and C2, 0.025 for C3) with four different baseline periods: the default 7 periods (weeks, here), plus 14, 28, and 56 weeks.

For the weekly statistical summaries, thresholds were calculated from the week of the year median, mean plus two standard deviations (Cullen method without removing past outbreaks), and 75th and 85th percentiles (WHO method) for three historical time periods: 5 years, 6 years, or weekly maximum of 6 or 7 years depending on the week of year.

The Farrington algorithm offers parameters to control various model settings, of which we focused on five: 1) the number of weeks before and after the week in question to use as a window for calculations (‘window half-size’, w), 2) the number of years of historical data to use (b), 3) the inclusion of an optional long-term trend, 4) the number of periods to account for seasonality, and 5) the number of weeks to exclude at the beginning of the evaluation period (for events that may already be in progress). For the Farrington algorithm, 204 variations in a parameter sensitivity analysis were run. The first four runs used basic settings without population offsets: 1) original method with no seasonality (one period), 2) original method with four periods for seasonality, 3) improved method with no seasonality, 4) improved method with four periods of seasonality. The original method was first proposed by Farrington et al. [28] and the improved method has the changes proposed by Noufaily et al. [29]. The improved method aims to reduce the number of false positives through changes in the calculations of the trend component, reweighting of past events, seasonality, and error structures [29]. The set of two hundred additional runs utilized the improved method with a population offset option and an exhaustive set of combinations of the selected five parameters and values: window half size (3, 5), years of historical data to include (3, 4, 5, 6, or maximum adaptive), long-term trend inclusion (trend or no trend), seasonality periods (1, 2, 4, 8, 12), and past weeks to exclude at the beginning for spin up time (26 or set equal to window half size). All parameter combinations can be found in the supplemental materials (Additional File 2, Supplemental Tables S1 and S2) and a subset of relevant parameter combinations in the Results (Table 4). All methods were implemented in R and the surveillance package was used for the EARS and Farrington methods [27, 45].

#### Skill comparison test

As a skill comparison test to the real detection algorithms, six sets of random alarms were also generated. Any algorithm that produces alarms will, by chance, occasionally occur during an event, and the more alarms triggered, the more likely events will seem to be detected. This skill test checked that the event detection methods are performing better than a null model and provided context in the comparison between the methods. The random algorithm produced alarms between one and five weeks long, with a minimum buffer of four weeks between alarms. The probability per week of an alarm was varied to create different total numbers of alarms.

#### Metrics

Metrics of detection effectiveness were event based, because using events as the unit of analysis is relevant to how these algorithms would be used in public health surveillance to find outbreaks before or as they are occurring. Two main indicators were used: the percent of events that were caught, and the percent of alarms that were associated with events (true positive alarms). An alarm and event were considered associated if the alarm was triggered any week during or up to two weeks prior to the event. Percent of events caught was an indicator of how well the algorithm detected events, with higher percentage meaning that fewer events were missed. Percent of alarms associated with events was the true positive rate of the alarms (the percentage of alarms that overlapped with or up to two weeks prior of an event). A high percentage of this metric demonstrated that the algorithm was more likely to trigger alarms when an event was actually happening and less likely to generate false alarms. Ideally, event detection algorithms would trigger alarms for all events (100% events detected) and never when there was not an event (100% alarms true positive). In addition to events caught, we also considered if the alarm for the event was timely, which was defined as an alarm between two weeks prior and including the start week of an event.

## Results

### Identified events

The TWST algorithm, developed to identify time periods of excess malaria case counts that were considered of potential public health interest, found a total of 255 events for *P. falciparum* and mixed species. The numbers of events declined from 2013 to 2018, however in 2019 the number of events greatly increased. Also, during 2019, the average number of cases in events was the highest since 2012 (Table 2, all events are shown over time in Additional File 1, Supplemental Fig. 1).

**Table 2:** Events and malaria case statistics for *P. falciparum* and mixed malaria

Year	Events	Average # of cases per event	Total # of cases during events	% of yearly cases during events
*2012 (W28-W52)*	*34*	*6500*	*220,983*	*72*
2013	53	2749	145,686	42
2014	52	1630	84,761	39
2015	32	2635	84,304	38
2016	34	1769	60,162	30
2017	15	1824	27,353	22
2018	5	2667	13,334	13
2019	30	2950	88,498	42

**Fig. 3:**
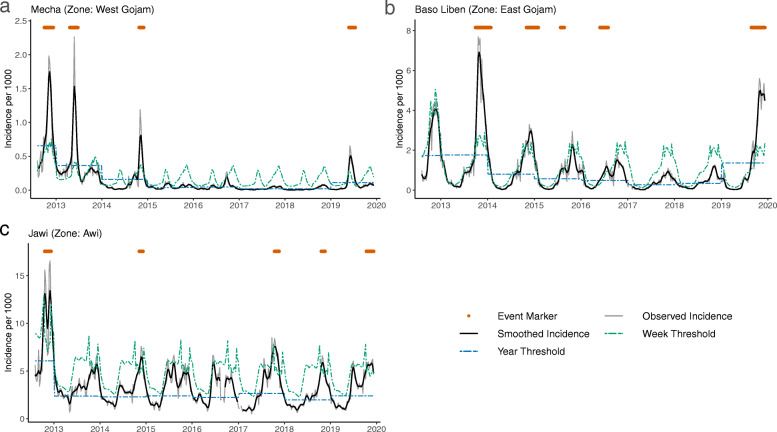
Malaria incidence and TWST-identified events in selected woredas. The examples show various patterns in seasonal and long-term trends in the incidence of malaria and the TWST events: (**a**) Mecha, (**b**) Baso Liben, (**c**) Jawi. Mecha and Baso Liben both had decreasing incidence and a resurgence in 2019, but Baso Liben had maintained seasonal peaks while Mecha did not. Seasonal patterns also vary between clear single or dual peaks to more irregular patterns such as in Jawi. Observed incidence is marked in light grey and the smoothed incidence in black. Week and year thresholds from the TWST algorithm are shown as dot-dashed lines in green and blue, respectively. Any identified events are marked with red circles at the appropriate weeks at the top of the graphs

The TWST algorithm was specifically designed to account for seasonality and not identify every seasonal peak as being an event in the context of overall declining trends in malaria transmission. However, different woredas in the region exhibited various patterns in incidence, including decreasing trends, increases in the middle or end of the time period, clear single seasonal peaks, dual seasonal peaks, and various combinations of these patterns. The TWST algorithm was flexible enough to appropriate identify events in these patterns (Fig. 3).

**Fig. 4:**
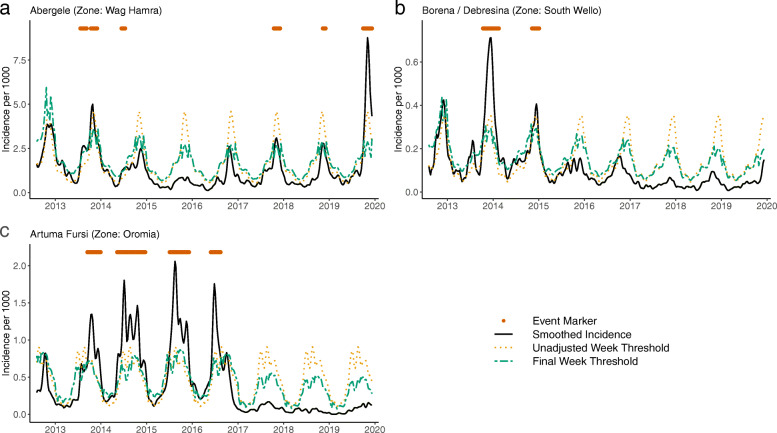
Example TWST results versus unadjusted week thresholds. Malaria incidence, unadjusted and adjusted week thresholds, and TWST events are shown for the selected woredas: (**a**) Abergele, (**b**) Borena / Debresina, and (**c**) Artuma Fursi. In Abargelie, the fall 2017 and 2018 peaks would have been below an unadjusted week threshold (orange dotted line) but were identified using the final TWST thresholds (green dot-dashed line) that had been trend-weighted. Borena / Debresina and Artuma Fursi shows multiple instances where the non-peak expansion and time-shift allowances prevented inappropriately identified events on the edges of incidence peaks or in the seasonal troughs

The algorithm was able to identify peaks that would have been overshadowed by peaks in much earlier years but are important relative to more recent patterns. For example, the woreda Abargelie had high peaks in 2013 and to a lesser extent in 2014. During 2015 however, the season was very quiet with no large peaks. In the fall of 2016, a moderate seasonal peak returned and larger fall peaks occurred in 2017 and 2018, but if the thresholds had not considered the 2015 season (trend-weighting), the 2017 and 2018 peaks would not been identified as events (Fig. 4). The time-shift allowance in TWST was also needed to prevent notifications of events where the peak simply declined more slowly than in other years (Fig. 4).

### Event detection

**Fig. 5:**
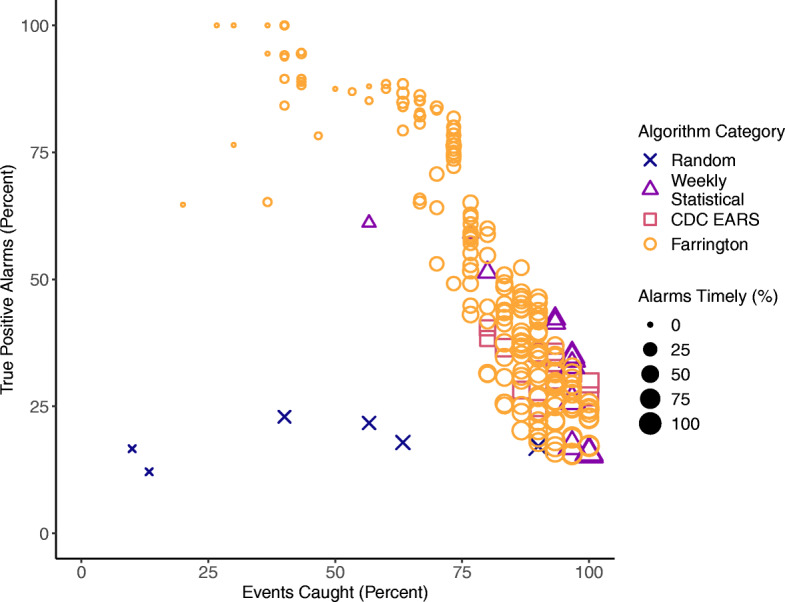
Scatter plot of the percent of events caught versus percent of true positive alarms. Results are shown from all event detection algorithm and variants. Each category is marked in a different shape and color combination. The size of the marker shows the percent of a timely alarms for an event

A total of 234 event detection algorithm and variations were tested on the 30 TWST-identified events in the 2019 evaluation period (selected entries in Table 3, for all results see Additional File 2, Supplemental Tables S1 and S2). Of the 234, there were 12 CDC EARS variants, 12 WHO and statistical variants, 205 Farrington variants, and six random alarm generators (Fig. 5). The six random alarm generators were run with various probabilities of generating an event per week: 0.2, 0.1, 0.05, 0.025, 0.012, and 0.006 which yielded 233, 151, 92, 61, 33, and 18 alarms respectively, a range similar to the number of alarms from the other event detection algorithms.

**Table 3:** Results for selected event detection algorithms for *P. falciparum* and mixed malaria events in 2019

Algorithm	% Events caught	% Timely	% True positive alarms	Total number of alarms
Random (*p* = 0.2)	90	70	17	233
Random (*p* = 0.05)	57	27	22	92
Random (*p* = 0.012)	13	3	4	33
EARS C1 (7 weeks)	90	53	25	146
EARS C2 (7 weeks)	100	73	27	152
EARS C3 (7 weeks)	100	87	30	135
WHO mean + 2sd (5 years)	80	11	51	72
WHO 75th percentile (5 years)	97	28	26	200
WHO 85th percentile (5 years)	97	73	35	143
Farrington A1	97	80	19	203
Farrington A2	100	87	25	163
Farrington B1	90	63	24	146
Farrington B2	93	63	29	116
Farrington C1	100	77	26	156
Farrington C2	40	7	100	16
Farrington C3	73	37	74	39
Farrington C4	83	53	51	63

Farrington parameter details can be found in Table 4

As expected, random alerts performed poorly and had the lowest percentages of true positive alarms across the variants (Table 3, Fig. 5). Variants with higher probabilities created more alarms, and saw higher event caught percent scores, as the more alarms are present the more likely they are to randomly overlap with an event.

The CDC EARS methods generated large numbers of alarms (98 to 152), with high percentages of events caught (80 to 100%) and a wide range of percent timely alarms (43 to 87%), but also had low to midrange percentages (25 to 40%) of true positive alarms (selected items in Table 3, full listing in Supplemental materials). Of the weekly statistical summaries, the Cullen mean plus two standard deviations variant produced the highest true positive rates (51 to 61%, depending on the number of years of historical data included), but the lowest percentages of events caught (57 to 80%) and the lowest percentages of timely alarms (13 to 37%). The WHO 75th percentile with 5 years of data, a commonly used algorithm, produced 200 alarms with 97% of events caught (93% timely) but only 26% true positive detections (Table 3). The 85th percentile variants produced somewhat fewer alarms with higher true positive rates, and with similar or slightly reduced events caught and timely alarms.

**Fig. 6:**
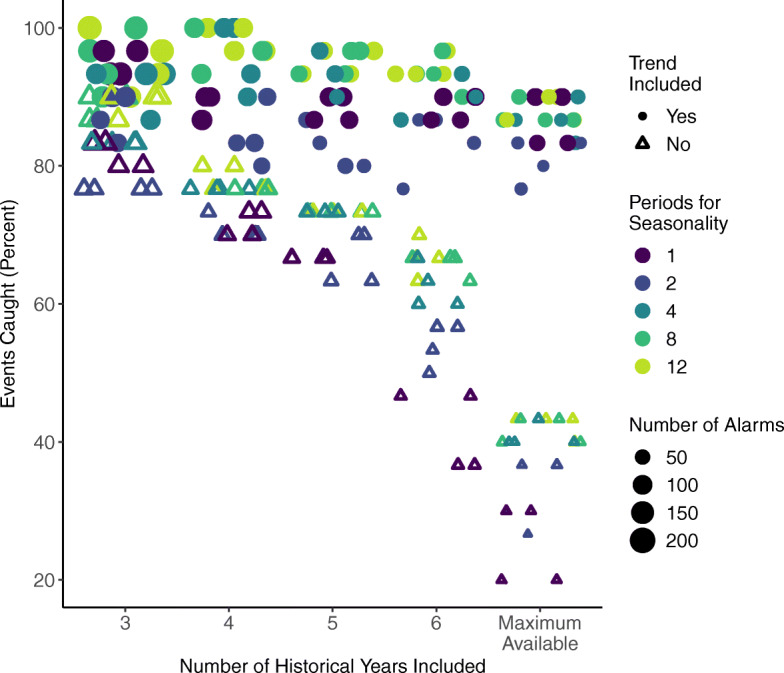
Plot of event caught percentages from the Farrington event detection variants. Scores were higher when a long-term trend was included (filled circles) than when no trend was included (hollow triangles). Event caught rate fell as more years of historical data were included (x-axis), especially in variants that did not include a trend. Within each trend set, scores were higher with 4 to 12 periods for seasonality (blue to green colors), and lowest with one period, i.e. no seasonality (dark purple). The number of alarms generated is indicated by the size of the marker and decreases as more years of historical data are included

Examining the Farrington results (orange hollow circles in Fig. 5), there was a trade-off between events caught and true positives. The Farrington variants were based on the sensitivity analysis of five parameters: window half size (w), years of historical data included (b), number of periods for seasonality, long-term trend inclusion, and the exclusion period for spin up time. Not all parameters influenced the outcomes; window half size and the exclusion period did not greatly affect the results, although the 26-week exclusion period was slightly preferable. The parameters for number of historical years of data, number of periods for seasonality, and trend inclusion had the greatest impacts on the outcome metrics. Of the 200 improved Farrington variants with population offset, the event caught rate was highest when the trend was included and there were 4 to 12 periods for seasonality (Fig. 6). The event caught rate fell as more years of historical data were included, especially in variants that did not include a trend.

**Fig. 7:**
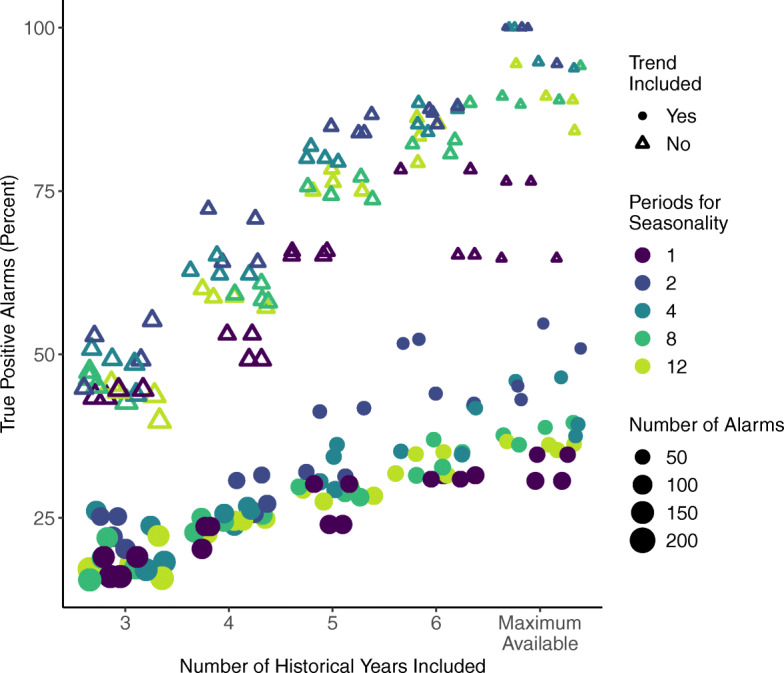
Plot of true positive alarm percentages from the Farrington event detection variants. Scores were higher when the long-term trend was not included (hollow triangles) as compared to variants where trend was included (filled circles). More historical data (x-axis) increased the alarm true positive score and decreased the total number of alarms generated (size of marker). Scores were highest with two to four periods for seasonality (blues), and lowest with no seasonality (one period, dark purple). The number of alarms generated is indicated by the size of the marker and decreases as more years of historical data are included

Of the 200 improved Farrington variants with population offsets, the true positive percentages were highest when no trend was included, two to four periods for seasonality were included, and increases as more years of historical data are included (Fig. 7). The number of alarms generated decreased with additional years of historical data (size of the marker in Figs. 6 and 7).

The Farrington original and improved methods with default values (and without seasonality) and no population offset were compared against the 200 parameter sensitivity runs using the improved method with population offsets (original A1 and A2, base improved B1 and B2 in Tables 3 and 4). As seen in Figs. 6 and 7, there were large trade-offs in the 200 variant set between events caught and true positive rates. Some Farrington runs reached 100% events caught, but the highest true positive rate of that set was only 26% (Farrington C1 in Table 3). Other variants reached 100% in alarm true positive rate, but the highest event caught score in that set was 40% (Farrington C2 in Table 3). Taking a balanced approach, a variant with reasonable trade-offs had a score of 73% events caught and 74% alarm true positive but only 37% events caught timely (Farrington C3 in Tables 3 and 4). Another, and our selected balanced variant had 83% events caught and 53% events caught timely with 51% alarms true positive (Farrington C4 in Tables 3 and 4).

**Table 4:** Details of selected Farrington variants, including parameter settings

Farrington Label	Description	Selected Parameters
A1	Original Farrington method, Base settings, No seasonality, No population offset	Window half size (w) = 3; Years (b) = 5; Seasonality periods = 1; Trend conditionally included (at 0.05 alpha threshold); Past weeks to exclude = w
A2	Original Farrington method, Base settings, Seasonality with four periods	Window half size (w) = 3; Years (b) = 5; Seasonality periods = 4; Trend conditionally included (at 0.05 alpha threshold); Past weeks to exclude = w
B1	Improved Farrington method, Base settings, No seasonality	Window half size (w) = 3; Years (b) = 5; Seasonality periods = 1; Trend included; Past weeks to exclude = 26
B2	Improved Farrington method, Base settings, Seasonality with four periods	Window half size (w) = 3; Years (b) = 5; Seasonality periods = 4; Trend included; Past weeks to exclude = 26
C1	Improved with population offset; *Highest true positive in highest caught set*	Window half size (w) = 5; Years (b) = 4; Seasonality periods = 4; Trend included; Past weeks to exclude = 26
C2	Improved with population offset; *Highest caught in highest true positive set*	Window half size (w) = 3; Years (b) = maximum; Seasonality periods = 8; Trend excluded; Past weeks to exclude = w
C3	Improved with population offset; *Balanced trade-off option*	Window half size (w) = 3; Years (b) = 5; Seasonality periods = 8; Trend excluded; Past weeks to exclude = w
C4	Improved with population offset; *Selected balanced trade-off*	Window half size (w) = 5; Years (b) = 3; Seasonality periods = 4; Trend excluded; Past weeks to exclude = w

## Discussion

The TWST algorithm that we developed succeeded in identifying malaria transmission events in the presence of changing expectations due to decreasing incidence trends. Using thresholds defined from time periods with high disease transmission may mask important events in less active years; events which would be considered abnormal if compared to more recent activity. This approach is essential in areas like the Amhara region where malaria incidence is declining in many woredas because of public health interventions. In regards to malaria surveillance, the WHO specifically notes that the normal or expected patterns of malaria, from which outbreak thresholds are derived, do change over time in areas that see sharp decreases in incidence after intensive control efforts [4]. As woredas approach elimination, the sizes of malaria events become smaller, but it will still be necessary to detect and respond quickly to these outbreaks. In the context of resurgence, having dynamic thresholds that adapt to changing conditions is crucial for identifying malaria peaks that are smaller than larger historical outbreaks, but still significantly larger than malaria case numbers in recent years.

The operational activities of detecting and responding to outbreaks are enabled by and integral to malaria surveillance systems. Surveillance as an intervention is the third pillar of the WHO global technical strategy for malaria elimination with differing key aspects as disease control transitions to pre-elimination, elimination, and prevention of reintroduction phases [7–9, 46–51]. More recent frameworks focus on transitions and evolving approaches needed in setting with changing epidemiology patterns [7, 46]. The Amhara region, as mentioned previously, is in a transition period marked by declining and changing trends in malaria transmission due to disease interventions plus a resurgence in 2019. By testing various algorithms on historical data from this region, we were able to assess their potential to provide early detection of resurgent malaria outbreaks. While weekly statistical methods are very commonly used for malaria surveillance [4], the CDC EARS and Farrington algorithms to our knowledge have not been previously assessed for use in malaria surveillance.

In the event detection comparison, the randomly generated alarms produced the worst results, indicating that all the algorithms that we tested were better than the naïve assumption of random outbreaks. CDC EARS is designed to be used even when lacking historical data, as it creates thresholds from only recent data (7 for C1 and C2 to 11 for C3 previous time steps with a baseline of 7). A drawback is that this approach cannot effectively account for seasonality and tends to trigger alarms at every seasonal peak. However, the results indicate that the EARS algorithms have a high sensitivity to increases in malaria cases. Thresholds based on weekly statistical summaries also produced high event caught scores and moderately higher alarm true positive rates as compared to CDC EARS methods. Both EARS and WHO methods tended to produce a high total number of alarms generated.

The suite of Farrington methods, especially the improved versions, allows adjustments for long-term trends and seasonal patterns. The Farrington algorithm, in various forms, have been implemented at public health centers and used for a variety of pathogens, particularly for gastrointestinal illnesses in several European countries: England, Wales, and Northern Ireland [28, 33], Scotland [24], Netherlands [24, 33, 35], Lower Saxony state in Germany [33], and Sweden [33, 34]. As with the weekly statistical summaries, the Farrington algorithms require several years of historical data, which may not always be available. As expected with the highly seasonal patterns we observe in the Amhara region, including enough seasonal periods was important as accuracy suffered when too few periods were included.

A substantial trade-off was found with the inclusion of long-term trend between the percent of events caught and the percentage of true positive alarms. Including the long-term trend as implemented in the Farrington algorithm increased events caught rate, however, there was also a decrease in the true alarm rate. In the context of declining malaria incidence, setting thresholds based on historical data tends to result in a high threshold that cannot detect smaller, more recent events. Adjusting the threshold using the recent trend of declining malaria cases therefore increases the sensitivity of outbreak detection but can result in large numbers of false alarms if the resulting threshold is too low. These results do show that accounting for annual cycles and inter-annual trends is essential for calibrating malaria early detection parameters in settings characterized by seasonal transmission and declining malaria trends caused by public health interventions. In situations where comprehensive data on interventions are available, other modeling approaches that explicitly account for interventions could also be used to predict trends in malaria cases [52].

One of our motivations for comparing early detection algorithms was to guide the selection of methods for a malaria early warning system in the Amhara region as part of the EPIDEMIA project [39]. Following discussions among project partners and in consideration of the public health applications of the early detection results, we opted then to give the true positive metric slightly more importance in the evaluation of algorithm performance. We did not want to generate large numbers of false alarms with an algorithm that had lower specificity, and we were cognizant that false alarms could cause ineffective and costly unnecessary mobilizations of resources. However, we balanced this desire to avoid false positives with the need to capture important events accurately and maintain credibility. In this analysis, we quantified the trade-off between events caught and true positive scores by testing a range of methods and parameterizations, and we found that variations of the Farrington method were usually best for maximizing both events caught and true positives.

Depending on the intended public health utilization of the event detection alarms, other implementations may choose to prioritize sensitivity over specificity if identifying all potential malaria outbreaks is more important than minimizing false positives. Methods and variants with high sensitivity could be useful for generating a ‘watch list’ of places that may be seeing an outbreak beginning or spike in cases. However, due to the high false alarm rate (low true positive percentage), warnings based on algorithm variants with low true positive scores run the risk of causing alert fatigue, where public health officials may be overwhelmed by alerts that are not meaningful. Alert fatigue has been observed with health care providers during health emergencies where they were inundated with public health communications and had trouble recalling specific information from the messages [53]. Health care providers tend to prefer fewer messages, from one source, and with local guidance or context [54]. Alarms from a system with high sensitivity but low specificity would not be suitable to prompt costly interventions, however, they may be useful to generate lists of places to monitor more closely.

Many of the early detection algorithms recommended for malaria use five full years to create the baseline. We tested five to 6.5 years in the weekly statistical summary methods, and from three to 6.5 years in the Farrington variants. However, given continuing changes in malaria transmission environments resulting from ongoing interventions, social and demographic changes, and climate change, it may not be reasonable to expect that historical malaria more than a few years old is suitable to provide a baseline for detecting future outbreaks [4, 25, 55–59]. Therefore, it is imperative to continue to explore new approaches for malaria outbreak detection that can be used with data covering shorter time periods. Future studies evaluating other algorithms will likely also prove insightful, as well as investigating the performance of the EARS and Farrington methods in other locations with different patterns of malaria incidence.

## Conclusion

We compared the effectiveness of three methods for malaria outbreak detection: 1) CDC EARS methods, 2) methods based on weekly statistical thresholds, including the WHO and Cullen methods, and 3) Farrington methods, using 7.5 years of malaria surveillance data from the Amhara region of Ethiopia. To our knowledge, this is the first study to assess the potential application of the EARS and Farrington methods for malaria outbreak detection. The EARS methods by design use a very short historical window that cannot account for seasonal trends in malaria occurrence. As a result, they were very sensitive to increases in cases and caught most outbreaks, but they could not effectively distinguish seasonal increases from outbreaks and generated many false positive alerts. WHO and statistical methods were also quite sensitive and detected high percentages of outbreaks with intermediate percentages of true positive alerts. Variations of the Farrington method had a wide range of trade-offs between events caught and true positive scores. Farrington variants that accounted for seasonality had much higher true positive rates than the EARS and WHO methods and could achieve a better balance between true positives and the percent of malaria events caught. We determined that of the methods compared, the Farrington algorithm was the most flexible and useful approach for operational early detection, and we have successfully used it in a pilot implementation of the EPIDEMIA malaria early warning system in the Amhara region [39]. We suggest that this approach is more generally useful for detecting infectious disease outbreaks in transitional environments with strong seasonality and declining trends. The intended used of the early detection results will drive the choice of algorithm and parameter settings to optimize sensitivity and specificity of alarms for particular applications.

## Supplementary Information

**Additional file 1. **Malaria events detected using the Trend Weighted Seasonal Threshold (TWST) algorithms. The file contains one figure (S1) for *Plasmodium falciparum* and mixed species of all events over time detected using the TWST algorithm in each of the 47 woredas.

**Additional file 2. **Variations of event detection algorithms tested. The file contains two tables for *Plasmodium falciparum* and mixed species of all the different parameter combinations tested. The first sheet is for the random, weekly statistical, and CDC EARS methods, the second sheet for the Farrington algorithms, and the third sheet for a description of the fields.

## Data Availability

The data that support the findings of this study are not publicly available because they were used under a data-sharing agreement with the Amhara Regional Health Bureau that does not permit their redistribution, but are available from the Amhara Regional Health Bureau on reasonable request. Code base for this project with synthetic data for demonstration can be found in a publically-available Github repository managed by our research group: https://github.com/EcoGRAPH/twst_event_detection_demo.

## Abbreviations

ARHB: Amhara Regional State Health Bureau
CDC: Centers for Disease Prevention and Control
EARS: Early Aberration Reporting System
EPIDEMIA: Epidemic Prognosis Incorporating Disease and Environmental Monitoring for Integrated Assessment
PHCU: Primary Health Care Units
TWST: Trend Weighted Seasonal Thresholds
WHO: World Health Organization
