# Supplementary material for: Comparing malaria early detection methods in a declining transmission setting in northwestern Ethiopia
Source: BMC Public Health. 2021 Apr 24;21:788. doi: 10.1186/s12889-021-10850-5 (PMC8067323; doi:10.1186/s12889-021-10850-5)
Supplement: Supplementary file 1 — Additional file 1. Malaria events detected using the Trend Weighted Seasonal Threshold (TWST) algorithms. The file contains one figure (S1) for Plasmodium falciparum and mixed species of all events over time detected using the TWST algorithm in each of the 47 woredas. [file 12889_2021_10850_MOESM1_ESM.pdf]

Malaria Early Detection in a Declining Transmission Setting in the Amhara Region of Ethiopia

Dawn M. Nekorchuk; Teklehaimanot Gebrehiwot; Mastewal Lake; Worku Awoke;

Abere Mihretie; Michael C. Wimberly

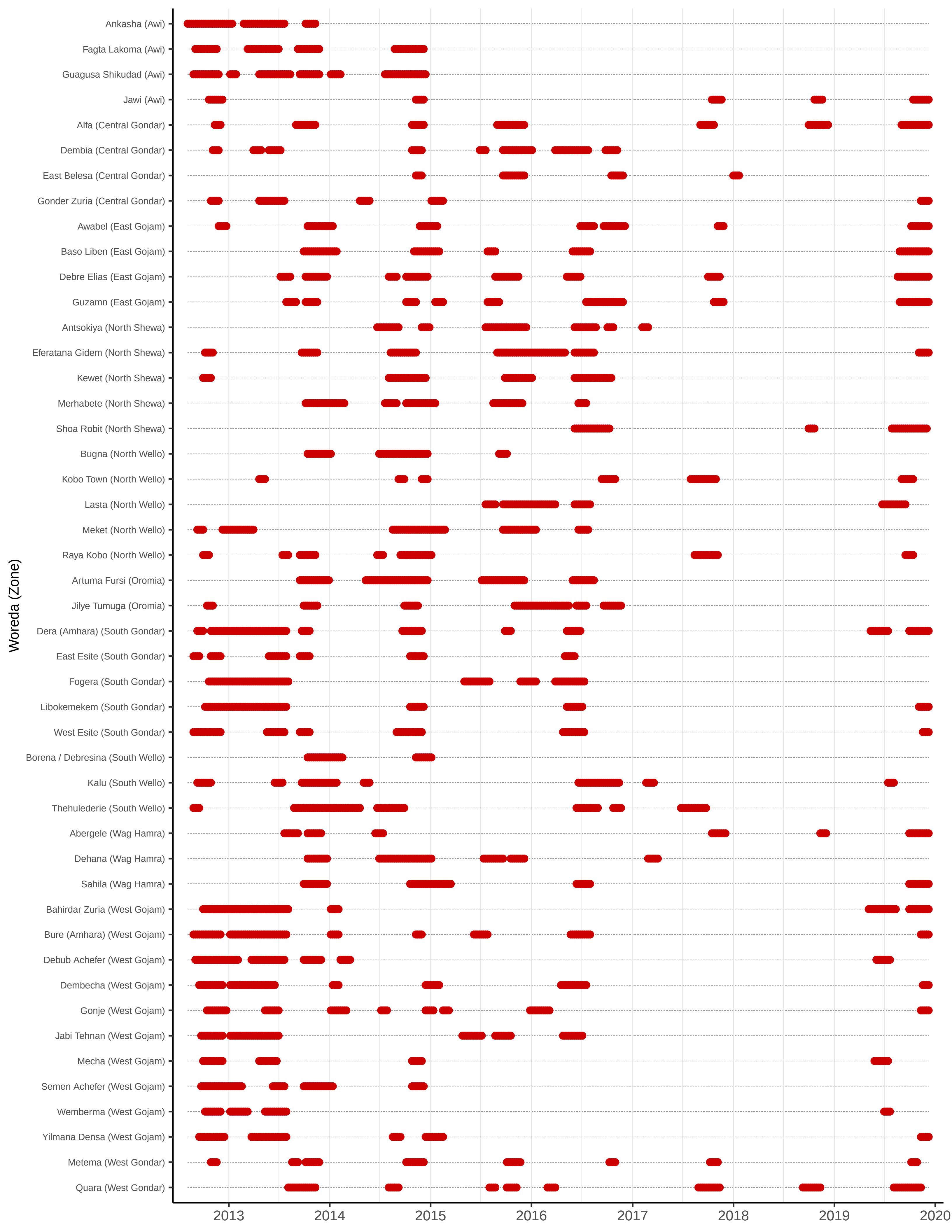
Woreda (Zone)
Ankasha (Awi)
Fagta Lakoma (Awi)
Guagusa Shikudad (Awi)
Jawi (Awi)
Alfa (Central Gondar)
Dembia (Central Gondar)
East Belesa (Central Gondar)
Gonder Zuria (Central Gondar)
Awabel (East Gojam)
Baso Liben (East Gojam)
Debre Elias (East Gojam)
Guzamn (East Gojam)
Antsokiya (North Shewa)
Eferatana Gidem (North Shewa)
Kewet (North Shewa)
Merhabete (North Shewa)
Shoa Robit (North Shewa)
Bugna (North Wello)
Kobo Town (North Wello)
Lasta (North Wello)
Meket (North Wello)
Raya Kobo (North Wello)
Artuma Fursi (Oromia)
Jilye Tumuga (Oromia)
Dera (Amhara) (South Gondar)
East Esite (South Gondar)
Fogera (South Gondar)
Libokemekem (South Gondar)
West Esite (South Gondar)
Borena / Debresina (South Wello)
Kalu (South Wello)
Thehulederie (South Wello)
Abergele (Wag Hamra)
Dehana (Wag Hamra)
Sahila (Wag Hamra)
Bahirdar Zuria (West Gojam)
Bure (Amhara) (West Gojam)
Debub Achefer (West Gojam)
Dembecha (West Gojam)
Gonje (West Gojam)
Jabi Tehnan (West Gojam)
Mecha (West Gojam)
Semen Achefer (West Gojam)
Wemberma (West Gojam)
Yilmana Densa (West Gojam)
Metema (West Gondar)
Quara (West Gondar)
2013
2014
2015
2016
2017
2018
2019
2020
